# Complete genome sequence of *Janthinobacterium kumbetense* LVF5 isolated from river freshwater

**DOI:** 10.1128/mra.00490-26

**Published:** 2026-07-22

**Authors:** Ines Friedrich, Jana Wagner, Anja Poehlein, Rolf Daniel

**Affiliations:** 1 Department of Genomic and Applied Microbiology, University of Göttingen 9375, Göttingen, Germany; University of Manitoba, Winnipeg, Canada

**Keywords:** *Janthinobacterium kumbetense*, violacein-producing bacteria, freshwater

## Abstract

We present the complete genome sequence of *Janthinobacterium kumbetense* LVF5, a violacein-producing bacterium isolated from freshwater of the Bremke River near Osterode am Harz, Germany. The genome consists of a single circular chromosome of 6,295,436 bp with a GC content of 62.91%.

## ANNOUNCEMENT

Members of the genus *Janthinobacterium* are known producers of violacein, an antimicrobial compound involved in microbial interactions (1). We report the complete genome sequence of strain LVF5, isolated from the Bremke River near Osterode am Harz, Germany (51°44′36.7″ N, 10°16′06.4″ E), on 12 December 2023, to expand the genomic representation of this genus. To enrich violacein-producing bacteria, cooked rice was used as a substrate following the method described by Corpe (2). Briefly, 1 mL of river water was added to a Petri dish (100 mm × 15 mm) containing cooked long-grain rice and incubated at 25°C. After 2 days, rice grains displaying dark-purple pigmentation were transferred to R2A agar plates (3). The isolate was repeatedly restreaked on R2A agar to obtain a pure culture and was designated LVF5. A single colony was inoculated into R2A broth and incubated overnight at 25°C and 180 rpm. Cells were harvested by centrifugation, and high-molecular-weight genomic DNA was extracted using the NucleoMag DNA Bacteria kit (Macherey-Nagel, Düren, Germany). Whole-genome sequencing was performed using Oxford Nanopore technology (Oxford Nanopore Technologies, Oxford, UK). For library preparation, 600 ng of genomic DNA was processed using the ligation-based Native Barcoding Kit 24 V14 (SQK-NBD114.24; barcode 16) following the manufacturer’s instructions (Oxford Nanopore Technologies). Genomic DNA was neither sheared nor size-selected prior to library preparation. Sequencing was carried out for 113 h employing a MinION Mk1B device equipped with an R10.4.1 flow cell and controlled by MinKNOW v25.05.12. Default settings were used for all software unless otherwise stated. Base calling and demultiplexing were performed using Dorado v1.2.0 (SUP model; Oxford Nanopore Technologies). Adapter and barcode sequences were trimmed during demultiplexing. Sequencing resulted in 173,058 reads representing 1,394,522,072 bp, with a read N50 of 11,826 bp. Reads shorter than 1 kb or of low quality were removed using Filtlong v0.3.1 (4). Filtered reads were subsampled with Trycycler v0.5.5 (5) using an estimated genome size of 6 Mb. Independent assemblies were generated with Flye v2.9.6 (6), Raven v1.8.3 (7), and Miniasm v0.3 (8) employing Minipolish v0.1.3 (7). Assemblies were combined using Trycycler and polished with Medaka v2.1.0 (Oxford Nanopore Technologies). Reads were mapped back to the final assembly using Minimap2 v2.29 (9), and mean coverage was calculated with SAMtools v1.22 (10), yielding 219-fold genome coverage. The final assembly consists of a single circular chromosome of 6,295,436 bp with a GC content of 62.91%. The genome was reoriented using Dnaapler v1.2.0 (11). Genome quality was assessed with CheckM2 v1.1.0 (12), and genome annotation was performed using the Prokaryotic Genome Annotation Pipeline (PGAP) v6.10 (13); the resulting genome features are summarized in Table 1.

**TABLE 1:** General features of the genome of *Janthinobacterium kumbetense* LVF5 (accession: JBWBFS000000000) based on PGAP annotation and CheckM2 analysis (12, 13)

Feature	Data
Total number of genes	5,685
Number of protein-coding sequences	5,562
Number of ribosomal RNA genes	
5S	9
16S	8
23S	8
Number of transfer RNA genes	94
Number of non-coding RNA genes	4
Number of pseudogenes	29
Genome completeness	100%
Genome contamination	0.1%

**Fig 1:**
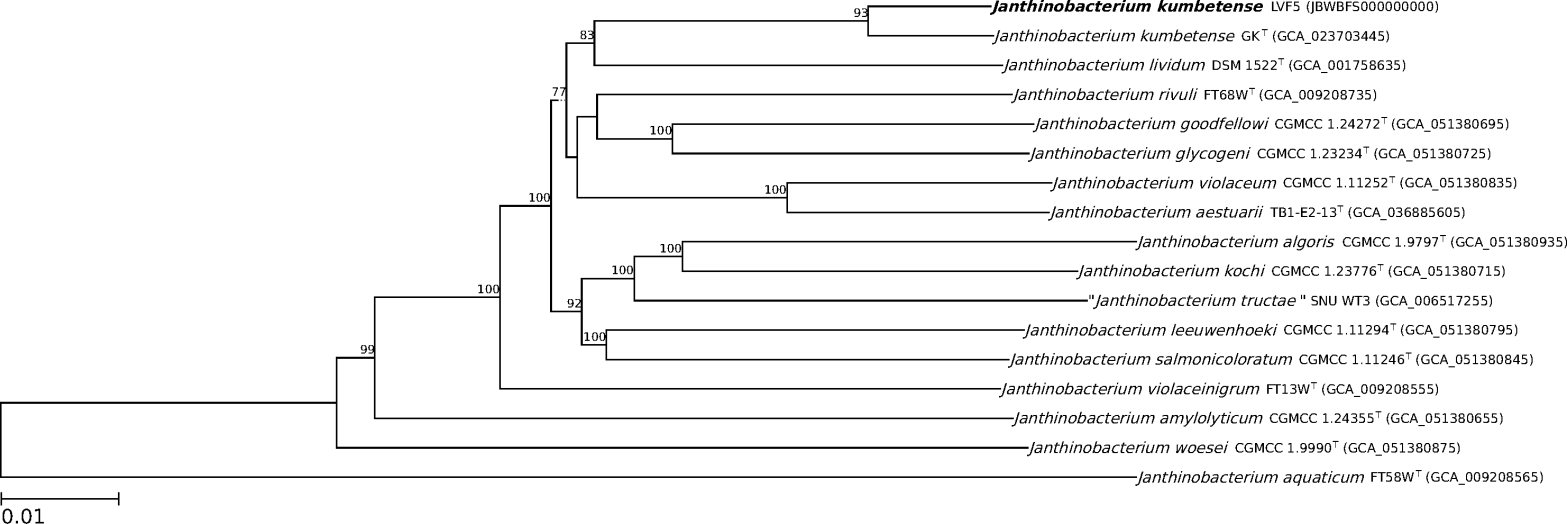
Whole-genome phylogenetic tree showing the position of *Janthinobacterium kumbetense* LVF5 (bold) among closely related *Janthinobacterium* type strains. The tree was inferred by using TYGS (14). The tree was calculated from genome BLAST distance phylogeny (GBDP) distances using FastME with 100 pseudo-bootstrap replicates (15). The tree was visualized using ETE 3 (16).

Whole-genome-based phylogenetic analysis of strain LVF5 was conducted using the Type (Strain) Genome Server (TYGS [14]; accessed 12 March 2026). The digital DNA–DNA hybridization value between LVF5 and *Janthinobacterium kumbetense* GK^T^ was 81.8% (formula d4), exceeding the species delineation threshold of 70%. The resulting phylogenetic tree is shown in Fig. 1. These results indicate that LVF5 represents a novel strain within the species *J. kumbetense*.

## Data Availability

The complete genome sequence has been deposited in DDBJ/ENA/GenBank under accession number JBWBFS000000000. Raw Nanopore reads are available in the National Center for Biotechnology Information Sequence Read Archive under accession number SRR37587131.
